# Tackling pressure fluctuations in ultra-HPLC to robustly resolve and analyze polar metabolites

**DOI:** 10.1016/j.jbc.2025.108283

**Published:** 2025-02-10

**Authors:** James R. Krycer, Manuel Plan, Thomas Stoll, Andrew R. Laskary, Mark P. Hodson, James E. Hudson

**Affiliations:** 1 QIMR Berghofer Medical Research Institute, Herston, QLD, Australia; 2 School of Biomedical Sciences, Faculty of Health, Medicine and Behavioural Sciences, The University of Queensland, St Lucia, QLD, Australia; 3 School of Pharmacy, Faculty of Health, Medicine and Behavioural Sciences, The University of Queensland, St Lucia, QLD, Australia; 4 School of Biomedical Sciences, Faculty of Health, Queensland University of Technology, Brisbane, QLD, Australia

**Keywords:** liquid chromatography–coupled mass spectrometry, analytical biochemistry, metabolism, Metabolomics, ultra-HPLC

## Abstract

The success of modern metabolomics analysis depends on the separation of metabolites in complex samples using methods such as LC and mass spectrometry. Herein, we present a protocol for resolving a broad range of polar metabolites, based on hydrophilic interaction LC with a zwitterionic-bonded phase (HILICz). In optimizing this protocol, we encountered pressure fluctuations, a widespread problem that impacts metabolite analysis, restricts batch sizes, and imposes instrument downtime, ultimately incurring substantial time and financial expense. Thus, we use this opportunity as a case study to demonstrate the steps taken to overcome such pressure fluctuations, resulting in a protocol that robustly and consistently resolves polar metabolites in large batches of samples (>100 samples, equating to >40 h of run time). This consistency is essential to address the growing demand for repeatable in-depth metabolomics analysis of complex samples.

An essential component of modern metabolomics is chromatography, which serves to separate metabolites to aid their detection and identification by mass spectrometry (MS). A popular method for resolving polar metabolites without the need for additional sample modification (*e.g.*, derivatization) is LC. LC aims to separate metabolites based on their interaction between the mobile phase (LC solvents) and stationary phase (particles contained within an LC column). There are a variety of LC chemistries available, each suited for particular classes of metabolites.

Here, we sought a methodology with broad coverage of polar metabolites that avoids ion-pairing reagents, which can contaminate all areas of the solvent flow path and create unwanted background for other methods used on the same LC instrument. Thus, we considered hydrophilic interaction LC with a zwitterionic-bonded phase (HILICz). This stationary phase has a high affinity for charged and polar molecules. Binding occurs using a mobile phase with a high proportion of organic solvent (*e.g.*, acetonitrile [ACN]) and elution by decreasing the organic solvent content (Fig. 1*A*). In particular, the Agilent InfinityLab Poroshell 120 HILICz column is reported to resolve a broad range of polar metabolites, when used with a mobile phase that is buffered by ammonium acetate (pH 9) ((1, 2), Fig. 1*B*). For instance, adenosine phosphates produced sharp peaks that elute in order of charge, that is, number of phosphate groups (Fig. 1*B*). Furthermore, this LC method could resolve metabolites that cannot be distinguished by MS/MS alone, including isobaric pairs such as glutamine–lysine (Fig. 1*B*) and succinate–methylmalonate (Table S1), where the *m/z* ratios of their precursor and preferred product ions are identical.

**Figure 1:**
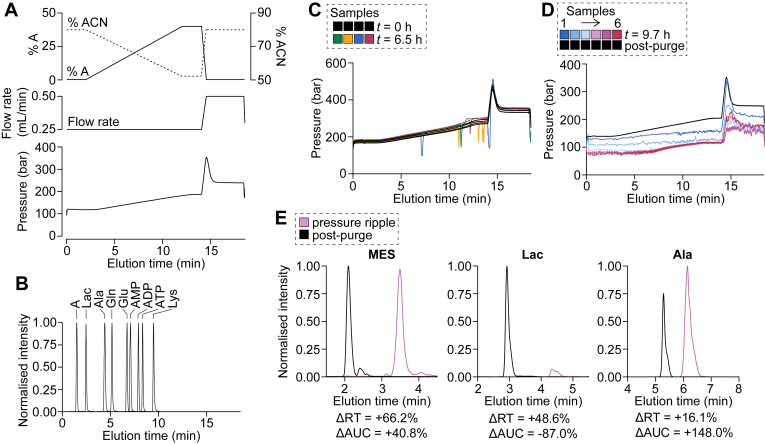
**Pressure fluctuations impact analyte retention times (RTs) and peak signal intensity.***A* and *B*, a mixture of standards, containing adenosine (*A*), lactate (Lac), alanine (Ala), glutamine (Gln), glutamate (Glu), AMP, ADP, ATP, and lysine (Lys), were subjected to the updated LC–MS protocol outlined in the *Experimental procedures* section and Table 1. Buffer A is mostly aqueous (solvent is 10% [v/v] acetonitrile in water), and buffer B is mostly organic (solvent is 80% [v/v] acetonitrile in water). The protocol involves a gradient from 0 to 40% (v/v) buffer A, followed by re-equilibration at 0% (v/v) buffer A (*A*, *upper panel*), with a flow rate of 0.25 ml/min except during the re-equilibration phase (*A*, *middle panel*). This produces a pressure trace dependent on the flow rate and proportion of buffer A (*A*, *lower panel*). In *B*, the intensity of metabolite peaks has been normalized to the maximum signal for each metabolite (their respective MS transition), overlaid to demonstrate the successful separation of these metabolites. *C*, cell culture media extracts (based on a matrix of DMEM) were subjected to the original LC–MS protocol. The pressure traces were generated from a subset of samples that were injected at the start (*t* = 0 h) and end (*t* = 6.5 h) of the sample batch. These samples exhibit normal pressure traces at *t* = 0 h (depicted by *black lines*) and pressure fluctuations at *t* = 6.5 h (depicted by *colored lines*, with each color depicting a different sample). *D*, cell culture media extracts (based on a matrix of DMEM) were subjected to the original LC–MS protocol. The pressure traces were generated from samples injected at run time ∼9.7 h (*colored lines*, with each color depicting a different sample), showing a complete deterioration in pressure trace with subsequent samples. The same samples were reinjected after purging the solvent lines (“postpurge,” depicted by *black lines*), showing restoration in the pressure traces. *E*, representative metabolite intensity chromatograms for sample 4 from *D*, with signal intensity normalized to the maximum intensity for the postpurge injection. Beneath each chromatogram, the change in retention time (RT) and peak area under the curve (AUC) from postpurge to prepurge (during pressure rippling) are shown. DMEM, Dulbecco's modified Eagle's medium.

However, upon following the manufacturer’s original HILICz protocol, we encountered substantial pressure fluctuations. This can indicate the presence of leaks, blockages, or air bubbles in the solvent lines. These issues are a common experience amongst LC users and can drastically impact the elution profile and signal of analytes. This not only impacts the analysis of individual samples but also introduces technical variability that prevents large sample batches and “high-end” techniques that only measure metabolites within specific time windows over an analytical run, such as dynamic (or scheduled) multiple reaction monitoring (MRM) in MS. Resolving pressure issues requires eliminating potential culprits by replacing LC components, such as pump seals, inline filters, and LC columns, ultimately incurring substantial time and financial expense.

Using our HILICz protocol as a case study, we outline steps that can be taken to overcome this issue, sharing our lessons in combatting pressure rippling and presenting an optimized LC protocol that robustly and consistently resolves a broad range of metabolite classes in large batches (>100) of samples.

## Results and discussion

### The pressure trace is a key diagnostic tool in LC

The “pressure” readout in LC specifically refers to back pressure caused by the LC tubing and column, measured as the decrease in pressure between the pump and distal end of the LC column. When an LC column is attached to the system, the pressure increases with the viscosity and flow rate of the mobile phase (since fluid flow is normally laminar as opposed to turbulent in nature) (3). Thus, our HILICz protocol normally elicits a rise in pressure over a single run because of: 1) the gradient of decreasing ACN content (increasing water content) throughout the run (Fig. 1*A*, *top panel*, 2–12 min), which would increase viscosity because ACN is less viscous than water (*e.g.*, μ = 0.35 mPa s for ACN *versus* 1.0 mPa s for water at 20 °C) and 2) the increase in flow rate during the re-equilibration at the end of the protocol (Fig. 1*A*, *middle panel*, 14–18.5 min). If these parameters are constant, the pressure should remain relatively stable (Fig. 1*A*, *bottom panel*, 0–2 and 12–14 min). Overall, the pressure trace is impacted by many LC parameters and thus provides an informative diagnostic readout that should be regularly monitored during LC analysis.

### Abnormal pressure traces indicate LC issues that can drastically impact metabolite analysis

When we initially used the HILICz LC column with a buffer system prepared according to the manufacturer’s instructions (1, 2), we encountered pressure fluctuations (“pressure rippling”) that manifested as either 1) sharp pressure fluctuations (Fig. 1*C*) and/or 2) an eventual degradation of the pressure trace (Fig. 1*D*). In both cases, these were not evident when the same samples were run at the start of the batch (Fig. 1*C*, *t* = 0 h *versus* 6.5 h) and were often eliminated by purging the LC lines (Fig. 1*D*, *cf* postpurge). This was concomitant with a shift in retention times and loss of peak signal (Fig. 1*E*, ΔRT and ΔAUC, respectively), the impact of which varied between metabolites. Thus, it is likely that the pressure rippling indicated disruptions to the LC system, which led to poor metabolite resolution, peak broadening, and ultimately loss in signal.

This was not isolated to culture media samples extracted with MeOH–ACN (Fig. 1, *C*–*E*)—we encountered similar issues with mouse plasma (extracted similarly) as well as mouse tissue (fecal, brain), organoids, and cell monolayers all extracted with MeOH–chloroform (4) (*data not shown*). Pressure rippling typically occurred within <10 h of run time (Fig. 1, *C* and *D*), ultimately restricting batch sizes to 10 to 15 samples, factoring in a run time of 20 min per sample, prerun equilibration, and the injection of blanks, calibration standards, and quality control samples to condition the column.

### Abnormal pressure traces can be caused by bubbles

In every case, there were no leaks, and solvent lines had been primed (“purged”) beforehand. The latter involves running solvent buffers through solvent lines at high flow rates (with flow diverted to waste instead of through the LC column) to equilibrate the solvent lines and remove air bubbles. We initially addressed the pressure rippling by repurging solvent lines. This only temporarily resolved the issue.

Next, we assumed that the pressure rippling was caused by bubbles formed by the mixture of organic and aqueous solutions, exceeding the capability of the degassing unit of our LC pump (Agilent part #G7120A). Thus, we

i. purged solvent lines with water, then isopropanol, then water prior to our mobile phases. Isopropanol is more viscous than water (*e.g.*, *μ* = 2.4 mPa s for isopropanol *versus* 1.0 mPa s for water at 20 °C) and can thus push bubbles from the LC tubing and capillaries more effectively than aqueous solutions;
ii. degassed mobile phases under vacuum with a sonicating water bath, prior to LC.

These steps extended the run time, but inevitably pressure rippling still occurred.

### Abnormal pressure traces can be caused by precipitation

We often observed precipitation visible in mobile phase buffer B, which could also cause pressure rippling. Precipitates would block the solvent path, being responsible for the rising pressure over time observed in our runs. As a temporary solution, we typically isolated the blockage by disconnecting each component of the LC path, for instance starting from the LC column and working backward to the pump. A sudden drop in pressure implicated the last component to be disconnected, which then needed to be unblocked and/or replaced.

The precipitation was likely because of the ammonium acetate in the presence of a high ACN content (90%, v/v). Thus, we mitigated this by

iii. reducing the ACN content of buffer B from 90% (v/v) to 80% (v/v). We subsequently added 10% (v/v) ACN to buffer A and adjusted the gradient to maintain the change in ACN content in the mobile phase over the run (Table 1, from 81 to 54% [v/v] ACN originally to 80% to 52% [v/v] ACN in the updated protocol). The former had the added benefit of improving the microbial resistance of buffer A.

**Table 1:** Comparison of buffer composition and gradient between the original and updated LC protocols

Parameter		Original protocol	Updated protocol
Mobile phases^a^			
Mobile phase A		100% H_2_O	10% ACN; 90% H_2_O
Mobile phase B		90% ACN; 10% H_2_O	80% ACN; 20% H_2_O


Gradient			
Time (min)	Flow (ml/min)	%A	%A
0	0.25	10	0
2	0.25	10	0
12	0.25	40	40
14	0.25	40	40
14.5	0.5	10	0
18.4	0.5	10	0
18.5	0.25	10	0

a All mobile phases include 10 mM ammonium acetate (set to pH 9 with NH_4_OH) and 5 μM medronic acid. Solvent proportions are presented as % (v/v).
iv. adjusting how buffer B was prepared. To generate 1 l of buffer B, we added (in the following order) 100 ml water, 100 ml of 100 mM ammonium acetate (adjusted to pH 9 with NH_4_OH), 0.5 ml of 10 mM medronic acid, then ACN in 100 ml increments (1–2 min apart), up to a final volume of 1 l. This was performed with constant stirring. Slowly introducing the ACN helped to minimize salt precipitation.
v. establishing a strict shelf-life for mobile phase buffers. While it is often recommended to prepare buffers as fresh as possible, it is common practice to use and even “top up” existing buffers, particularly in academic laboratories where funding availability necessitates pragmatic economic approaches to reduce waste. Using the original protocol, we observed that the precipitation in buffer B worsened over several weeks. This was accompanied with a drop in pH over time, presumably because of the volatility of NH_3_ in solution. Thus, to minimize precipitation and LC retention time drift, we adhered to a 1-week shelf-life for mobile phase buffers.
vi. washing the LC line and column with methanol/water (1:1), especially prior to storage. ACN is known to polymerize, particularly under high pressure (5), forming deposits that may cause pressure rippling. This can be mitigated by washing with buffers with a high-water content and increasing the water content of mobile phases that contain ACN.

### A robust LC protocol that can resolve a broad range of polar metabolites

**Figure 2:**
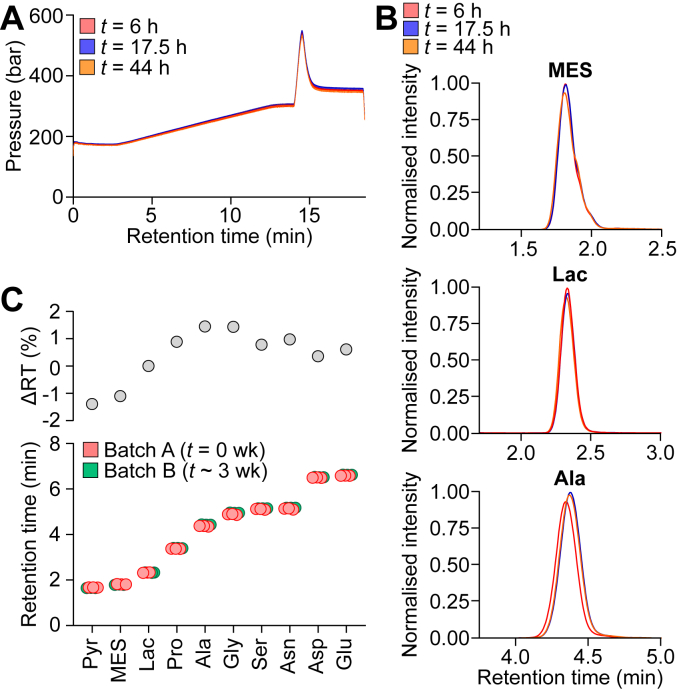
**The updated HILICz protocol robustly resolves a broad range of metabolites over an extended run.***A* and *B*, a mixture of neat standards (analytes listed in Table S1) was serially diluted one-in-three to generate a 10-point calibration curve. This set of standards was injected at multiple time points within the same LC–MS batch, subjected to the updated LC–MS protocol (Table 1). Pressure traces for each batch (*A*) are shown as mean ± SD. Representative metabolite intensity chromatograms from the second-highest calibration standard are shown in *B*, with signal intensity normalized to the maximum intensity across all three injections. Metabolite concentrations, as well as data for other metabolites, are presented in Table S1. *C*, approximately 3 weeks later, another calibration curve (containing the presented metabolites) was prepared and injected thrice, subjected to the same protocol as in *A* and *B*. The retention time (RT) for each individual injection (*lower panel*) and average change in RT (*upper panel*) are depicted.

**Figure 3:**
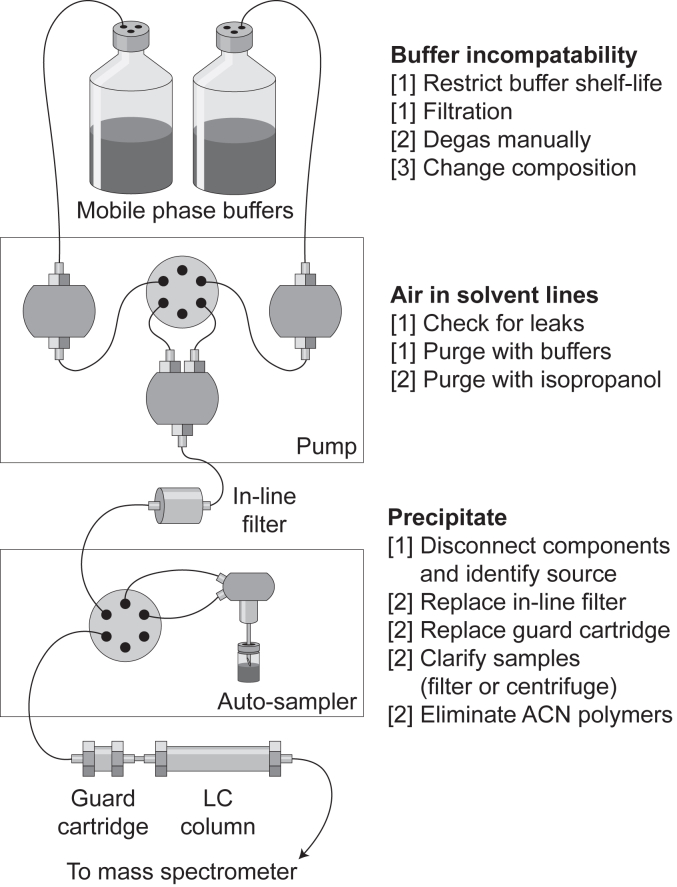
**Sources of pressure fluctuations in LC.** Steps to remedy these issues have been outlined in the main text and here have been triaged (1–3) based on effort cost. ACN, acetonitrile.

In troubleshooting this methodology, our optimized HILICz protocol exhibited stable pressure traces (Fig. 2*A*) and metabolite elution profiles (Fig. 2*B*) after ∼40 h of continuous run time. This protocol resolves polar metabolites spanning a range of chemical classes and polarities (including sugar phosphates, amino acids, organic acids, and nucleotides), with negligible drift in retention time when using fresh LC buffers prepared several weeks apart (Fig. 2*C*, Table S1). Last, we have used the same LC column for this protocol for >10 months and >2000 runs, practically doubling the lifespan of our previous HILICz LC columns. Together, pressure fluctuations are caused by multiple culprits across the LC system, all of which can adversely impact metabolite analysis (Fig. 1). Nevertheless, by following a triage of troubleshooting steps (Fig. 3), these can be eliminated to maximize the efficiency and robustness of metabolite resolution (Fig. 2). In addition to monitoring pressure traces, we recommend including pooled quality-control samples to monitor metabolite retention times and instrument response across the run (6).

Such LC stability is vital for advanced analytical techniques such as dynamic (scheduled) MRM, where metabolites (specifically, their reactions or transitions in the MS) are monitored within specific time windows instead of across the entire analytical run. This enables the MS to monitor fewer metabolites at a time (increasing the “dwell time” for each reaction), enhancing detection sensitivity and enabling up to hundreds of transitions to be monitored across a run. Thus, monitoring and tackling LC pressure issues is key to maximizing both LC and MS capability.

## Experimental procedures

### Ethics

Ethical approval for the use of human embryonic stem cells (hESCs) was obtained from QIMR Berghofer’s Ethics Committee and was carried out in accordance with the National Health and Medical Research Council of Australia regulations, abiding by the Declaration of Helsinki principles.

### Metabolite extraction from cell culture media

Conditioned cell culture media samples were derived from hESC-derived cardiac organoids, which were generated as described previously (7, 8). hESCs utilized were HES3 (WiCell) and CW30382A (CIRM hPSC Repository funded by the California Institute of Regenerative Medicine). Quality control was performed by short tandem repeat profiling, karyotyping, and mycoplasma testing.

Metabolites were extracted from conditioned media as described previously (4), with minor modifications. Specifically, media samples were combined with 4 volumes of extraction buffer containing 1:1 (v/v) mixture of methanol and ACN, with 1 μM 4-morpholineethanesulfonic acid (Sigma–Aldrich) as an internal standard. The mixture was vortexed briefly and centrifuged for 20 min at 16,000*g* and 4 °C. The supernatant was transferred into HPLC vials. Calibration standards were diluted in naïve media and then extracted in parallel to the samples.

### LC–MS

#### Overview

Metabolites were resolved by LC using a 1290 Infinity II pump (Agilent) with an InfinityLab Poroshell 120 HILIC-Z column (Agilent; 2.7 μm particle size, 2.1 mm internal diameter x 100 mm length, either PEEK-lined or with a guard column attached). The buffer systems and LC gradients are detailed in Table 1. To minimize pressure fluctuations in the optimized protocol, buffer B was prepared as outlined in the *Results* section—briefly, the aqueous components were mixed first, ACN was then added stepwise, and the buffer was degassed under vacuum prior to use.

#### LC passivation

To improve sensitivity and reduce peak-tailing, medronic acid was included as an additive (1). In conjunction, the LC line from the pump to the column (*i.e.*, detached from the MS) was passivated with phosphoric acid prior to use: the LC line was initially washed with water at 0.25 ml/min for 30 min, 0.5% (v/v) phosphoric acid in ACN–water (90:10) at 0.1 ml/min for at least 12 h, then with water at 0.25 ml/min for at least 1 h, ensuring the pH >4 by the conclusion of washing.

#### LC pretreatment and post-treatment

Prior to sample analysis, the LC column was conditioned with initial LC conditions (0% A) at 250 μl/min for 20 min, followed by at least three injections with water/ACN (1:1) and five “dummy sample” injections (typically the standard curve). After the batch was analyzed, the LC column was washed prior to storage using buffers wash A (water/methanol, 1:1) and wash B (ACN): 40% wash A for 10 min, 100% wash A for 30 min, then 20% wash A for 10 min, all at 250 μl/min. This ensured initially washing with 80% (v/v) organic solvent to match the pre-equilibration ACN content (Fig. 1*A*), 50% (v/v) methanol to eliminate any polymerized ACN, then 90% (v/v) organic solvent for storage.

#### Sample analysis

During sample analysis, the autosampler temperature was 4 °C, column temperature was 30 °C, and injection volume was 3 μl. MS analysis was performed using an Agilent 6470 QQQ with Jet Stream Technology Ion Source, with the following parameters: gas temperature at 200 °C, gas flow at 11 l/min, nebulizer pressure at 40 psi, sheath gas temperature at 400 °C, sheath gas flow at 12 l/min, capillary voltage at 3000 V for both negative and positive modes, and nozzle voltage at 0 V for negative mode and 500 V for positive mode. MRM transitions were calibrated and optimized using metabolite standards, with transition parameters available in Table S1 and upon request. Acquisition was performed with a 10 ms dwell time (standard MRM mode) or a cycle time of 500 ms (dynamic MRM mode). LC–MS data were extracted using MassHunter Qualitative Analysis (Agilent) and Skyline (9).

## Data availability

Unless otherwise indicated, the data described here are contained within the article. Unpublished data can be shared upon request by contacting the corresponding author (J. E. H., james.hudson@qimrberghofer.edu.au).

## Supporting information

This article contains supporting information.

## Conflict of interest

The authors declare that they have no conflicts of interest with the contents of this article.
